# The New Commissioner of Agriculture

**Published:** 1881-07

**Authors:** 


THE NEW COMMISSIONER OF AGRICULTURE.—There has been a most uniform commendation of the course of the President in appointing Dr. Loring Commissioner of Agriculture. Dr. Loring was the man who first cleared Massachusetts of pleuro-pneumonia, an act which alone would entitle him to the highest public gratitude and respect.

We believe that veterinarians will heartily join in the general approval of this appointment. From our knowledge of the interest which the new Commissioner takes in veterinary science, we feel sure that he will do what is possible to secure its advancement, appreciating, as he must do, its importance to the agricultural and commercial interests of the country.

We may reasonably expect that the efforts at investigating contagious diseases will be more intelligently directed than has heretofore been done. It is certain that the wretched stuff put forth as pathological work, by the late Commissioner, cannot be imposed upon his successor.

Every one will now be ready with suggestions for the new officer. One of these is, that he establish a Veterinary Bureau which shall investigate contagious diseases among animals, and which shall organize the means by which a knowledge of the prevalence of these contagious diseases can be obtained. Bulletins from such a Bureau would have authority and value beyond any possible unofficial announcements.

